# Synthesis and In Vivo Evaluation of Insulin-Loaded Whey Beads as an Oral Peptide Delivery System

**DOI:** 10.3390/pharmaceutics13050656

**Published:** 2021-05-04

**Authors:** Joanne Heade, Fiona McCartney, Miguel Chenlo, Olga Moreno Marro, Maja Severic, Robert Kent, Sinead B. Bleiel, Clara V. Alvarez, Brendan T. Griffin, David J. Brayden

**Affiliations:** 1 UCD School of Veterinary Medicine and UCD Conway Institute, University College Dublin, Belfield, Dublin 4, Ireland; joanne.heade@ucdconnect.ie (J.H.); fiona.mccartney@ucd.ie (F.M.); 2 Insucaps LTD., 11 Herbert Street, Dublin, Dublin 2, Ireland; sinead.bleiel@anabio.ie; 3 Neoplasia & Endocrine Differentiation Group, Centre for Research in Molecular Medicine and Chronic Diseases (CIMUS), Campus Vida, University of Santiago de Compostela, 15782 Santiago de Compostela, Spain; miguelangel.chenlo@usc.es (M.C.); clara.alvarez@usc.es (C.V.A.); 4 AnaBio Technologies Ltd., IDA Business Park, Co. Cork, T45 RW24 Carrigtwohill, Ireland; olga.moreno@anabio.ie (O.M.M.); maja.severic@anabio.ie (M.S.); robert.kent@anabio.ie (R.K.); 5 School of Pharmacy, University College Cork, T45 RW24 Cork, Ireland; brendan.griffin@ucc.ie

**Keywords:** insulin, whey protein isolate, oral peptide delivery, encapsulation, diabetes, intestinal permeability

## Abstract

For many diabetics, daily, lifelong insulin injections are required to effectively manage blood glucose levels and the complications associated with the disease. This can be a burden and reduces patient quality of life. Our goal was to develop a more convenient oral delivery system that may be suitable for insulin and other peptides. Insulin was entrapped in 1.5-mm beads made from denatured whey protein isolate (dWPI) using gelation. Beads were then air-dried with fumed silica, Aerosil^®^. The encapsulation efficiency was ~61% and the insulin loading was ~25 µg/mg. Dissolution in simulated gastric-, and simulated intestinal fluids (SGF, SIF) showed that ~50% of the insulin was released from beads in SGF, followed by an additional ~10% release in SIF. The omission of Aerosil^®^ allowed greater insulin release, suggesting that it formed a barrier on the bead surface. Circular dichroism analysis of bead-released insulin revealed an unaltered secondary structure, and insulin bioactivity was retained in HepG2 cells transfected to assess activation of the endogenous insulin receptors. Insulin-entrapped beads were found to provide partial protection against pancreatin for at least 60 min. A prototype bead construct was then synthesised using an encapsulator system and tested in vivo using a rat intestinal instillation bioassay. It was found that 50 IU/kg of entrapped insulin reduced plasma glucose levels by 55% in 60 min, similar to that induced by subcutaneously (s.c.)-administered insulin (1 IU/kg). The instilled insulin-entrapped beads produced a relative bioavailability of 2.2%. In conclusion, when optimised, dWPI-based beads may have potential as an oral peptide delivery system.

## 1. Introduction

The vast majority of marketed peptides are administered via injection [1], which is not a convenient route for patients, especially those suffering from chronic illnesses requiring daily injections. Subcutaneous (s.c.) injections are also not the safest route for some peptides, like insulin, which is associated with many adverse effects due to the unphysiological nature of this delivery route [2]. Oral delivery is by far the preferred route of patients, but it is difficult to achieve for peptide formulations because peptides are subjected to proteolysis throughout the gastrointestinal (GI) tract and are often too hydrophilic and have too high a molecular weight to cross the intestinal epithelium in concentrations sufficient to produce a therapeutic response [3].

Several strategies can be used to tackle the obstacles associated with oral peptide delivery, and recent successes in achieving approvals for oral formulations of semaglutide and octreotide have encouraged efforts with other peptides. Oral semaglutide (Rybelsus^®^, Novo Nordisk, Bagsværd, Denmark), a glucagon-like Peptide-1 (GLP-1) analogue for the treatment of type 2 diabetes (T2D), was co-formulated with a permeation enhancer (PE), sodium salcaprozate (SNAC). It achieved a relative oral bioavailability of ~0.4–1.0% via gastric absorption in humans [4]. Oral octreotide (Mycapssa^™^, Chiasma Pharma, Jerusalem, Israel), a somatostatin analogue used for the treatment of acromegaly, also employs PEs to aid intestinal absorption. The formulation is an enteric-coated capsule containing octreotide and sodium caprylate (C_8_) and other excipients in a water-in-oil suspension and it yields an oral bioavailability of ~0.7% in humans [5]. Despite such low oral bioavailability, both oral formulations are effective due to their high potency and the structural modifications that increase their half-life (t_1/2_). Other strategies for oral peptide delivery include micro and nanoparticle carries for protection and controlled release, enzyme inhibitors to prevent degradation, and microneedle-based devices to bypass the intestinal epithelium, reviewed in [6].

In the current study, we selected human insulin as an initial model peptide to assess in a new delivery system. Insulin is a suitable candidate because in vitro and in vivo analytical assays are well-established and can be used to benchmark against other delivery technologies. An oral delivery system also allows insulin physiological access via the hepatic portal vein to the liver [7]. Insulin administered by the s.c. route can cause hypoglycaemia, while hyperlipidaemia is associated with injection-site reactions [8]. These adverse reactions can potentially be avoided by oral administration. On the contrary, insulin is a low therapeutic index molecule and is regarded as a high-risk medicine [9], so there could be safety issues with a sub-optimal oral system that failed to deliver reproducibly. Moreover, as there are insulin receptors in the GI tract, depositing high doses of insulin in the lumen might be associated with cell growth [10]. Still, modified basal insulin recently achieved 1–2% relative oral bioavailability in Phase II trials using a tablet comprising the PE, sodium caprate [11]. Though this value was higher than that achieved by Rybelsus^®^ and Mycapssa^®^, it was deemed by Novo Nordisk to be not commercially viable. With those caveats, we selected insulin as a model peptide and sought to use food-derived natural polymeric materials to deliver it across the GI tract because of their biocompatibility, biodegradability, and low cost [12]. Polymers for consideration included chitosan, alginate, starch, pectin, casein, gelatin, and whey protein [13].

Whey protein is a by-product of cheese manufacturing and has many properties that could be beneficial for oral insulin delivery. Denatured whey protein isolate (dWPI) has been shown to inhibit the intestinal peptidases trypsin and chymotrypsin to a greater extent than its native form (nWPI) [14], even when formulated into microparticles [15]. Such protease inhibition by dWPI likely occurs through competitive inhibition since β-lactoglobulin, the primary component of WPI is a substrate for both enzymes [16]. Whey protein is also a source of dipeptidyl peptidase-4 (DPP-4) inhibitory peptides, which can lower blood glucose by preventing the degradation of endogenous GLP-1 [17]. Permeation enhancement across the intestinal epithelium is also a reported property of dWPI. Déat-Lainé et al. showed a decrease in TEER and an increase in the Papp of blue dextran transport across Caco-2 monolayers in the presence of dWPI [18]. That study also showed small increases in the Papp of insulin in the presence of dWPI when insulin was encapsulated in WPI/alginate microparticles. Finally, WPI is also reported to be mucoadhesive, which might be beneficial for oral insulin delivery as it may increase formulation residence time in the small intestine to enable contact with the epithelium [19].

In this study, we used dWPI as an encapsulation matrix for insulin to form beads. We examined the basic characteristics of the insulin-dWPI formulation along with the effects of the drying method on bead characteristics. We also investigated the capacity of beads to protect insulin from protease degradation under in vitro simulated intestinal conditions, and the bioactivity of bead-released insulin in insulin receptor-expressing liver cells. We then scaled up the process by moving from manually synthesised beads to encapsulator-generated ones and used these for in vivo GI instillation study using normal rats where plasma glucose reductions could be demonstrated.

## 2. Materials and Methods

### 2.1. Materials

BiPro WPI (97%), was obtained from Davisco Foods International Inc. (Le Sueur, MN, USA). Gibco’s recombinant human insulin was purchased from ThermoFisher Scientific (Dublin, Ireland). Aerosil^®^-200F was a gift from Evonik Industries (Essen, Germany). HepG2 cells were obtained from the European Collection of Authenticated Cell Cultures (ECACC, Public Health England, Porton Down, UK) and used at passages 9–12. The plasmids, pSynSRE-T-luc and pSynSRE-Mut-T-luc, were purchased from Addgene (Cambridge, MA, USA), and pRSV-βgal was obtained from Promega (Madison, WI, USA). All other reagents were purchased from Sigma Aldrich (Arklow, Co. Wicklow, Ireland).

### 2.2. Preparation of Manual dWPI Beads Entrapped with Insulin

WPI was prepared by dissolving WPI (11% *w/v*) in water WPI was then denatured (dWPI) in a water bath at 78 °C for 45 min. The dWPI was used over 2 days and was made fresh every week. Insulin was dissolved in HCl (0.1 M) and the pH was adjusted to pH 7.4 using 4-(2-hydroxyethyl)-1-piperazine ethane sulfonic acid (HEPES). Dissolved insulin was combined with dWPI to create a 4% (*w/w*) insulin solution. The dWPI-insulin mixture was stirred gently for 15 min and then added dropwise using a 30G needle to a hardening solution made from an 0.5 M acetate buffer. The beads were cured for 15 min before being rinsed with water and air-dried with or without fumed silica, Aerosil^®^-200F (Evonik Industries, Essen, Germany). Dry beads were then sized using an Android Google Pixel 4 smartphone and ImageJ^®^ software (U.S. National Institutes of Health, Bethesda, MD, USA.

### 2.3. Insulin Loading and Encapsulation Efficiency in dWPI Beads

Insulin-dWPI beads were ground using a mortar and pestle and added to 25 mL Simulated Intestinal Fluid (SIF, pH 6.8) made according to United States Pharmacopeia (USP) specifications [20]. This solution stirred vigorously for 4–6 h until the beads were completely dissolved. 500 µL samples were then centrifuged at 10,000 rpm for 2 min using centrifuge filter tubes with a cellulose acetate membrane comprising 0.22 µm pores (Corning^®^ Costar^®^ Spin-X^®^) to separate insulin from fragments of dWPI. Insulin sample analysis was carried out using reverse-phase high-performance liquid chromatography (RP-HPLC). This was carried out on an Agilent 1200 series HPLC using an InfinityLab Poroshell 120 SB-C_18_ column (4.6 × 100 mm, 2.7 µm, Agilent, Cheadle, UK). Gradient elution was used with the mobile phases consisting of (A) water + TFA (0.1% *v/v*), and (B) acetonitrile. The gradient was set up as follows: 70:30 (0 min), 40:60 (5 min), and 70:30 (10 min). Samples were injected at a flow rate of 1 mL/min and the injection volume was 20 µL. Separation was carried out at room temperature and insulin was detected at a wavelength of 214 nm. Insulin standards were included in every run to ensure the quality of the data was maintained. This method was adapted from Sarmento et al. [21]. Encapsulation efficiency (EE), percentage loading, and final loading of insulin were then calculated using a standard curve by following Equations (1)–(3) respectively.

(1) $$\mathrm{EE}\;\left(\%\right)\;=\frac{\mathrm{Total}\;\mathrm{encapsulated}\;\mathrm{insulin}\;\left(\mathrm{mg}\right)}{\mathrm{Total}\;\mathrm{insulin}-\mathrm{waste}\;\left(\mathrm{mg}\right)}\times 100$$

(2) $$\mathrm{Loading}\;\left(\%\right)\;=\frac{\mathrm{Total}\;\mathrm{encapsulated}\;\mathrm{insulin}\;\left(\mathrm{mg}\right)}{\mathrm{Total}\;\mathrm{beads}\;\left(\mathrm{mg}\right)}\times 100$$

(3) $$\mathrm{Final}\;\mathrm{loading}\;\left(µg/\mathrm{mg}\right)\;=\frac{\mathrm{Total}\;\mathrm{encapsulated}\;\mathrm{insulin}\;\left(µg\right)}{\mathrm{Total}\;\mathrm{beads}\;\left(\mathrm{mg}\right)}$$

where total insulin is defined as the quantity of insulin mixed with the dWPI before encapsulation, and the waste is defined as the insulin content of any dWPI-insulin solution remaining after bead production (i.e., the residue inside the syringe or beaker).

### 2.4. Insulin Release Studies in Simulated Buffers

Insulin release from the dWPI beads was monitored in Simulated Gastric Fluid (SGF, pH 1.2) made according to United States Pharmacopeia (USP) specifications, and also in Simulated (small) Intestinal Fluid (SIF, pH 6.5) [20]. Using a paddle stirrer at 200 rpm, beads were stirred for 1 h in SGF and then transferred to SIF for a further 3 h. 500 µL samples were withdrawn at set time points into centrifuge filter tubes (0.22 µm pore size), centrifuged at 10,000 rpm for 2 min, and analysed by RP-HPLC. Release data were fitted to mathematical drug release models including zero-order, first order, Higuchi, and Korsmeyer-Peppas using the Microsoft^®^ Excel add-in software, DDSolver [22]. All equations used can be found in Supplementary Table S1. To further examine the mechanism of release, the swelling behaviour of insulin-loaded beads was investigated, and the protocol is shown in Supplementary Materials.

### 2.5. Circular Dichroism

The circular dichroism (CD) spectra of native and bead-released insulin were obtained using a Jasco J-810 spectropolarimeter (Jasco, Dunmow, UK). The parameters used were as follows: wavelength range: 190–400 nm; sensitivity: 100 mdeg; data pitch: 0.5 nm; bandwidth: 2 nm; response: 4 s; scanning speed 50 nm/min; accumulations: 4; temperature: room temperature (20 °C). Insulin was released from beads into SIF and the insulin concentration was measured by HPLC. Insulin samples were diluted to 10 µg/mL in SIF before taking measurements. A quartz cuvette (Starna Scientific^®^, Essex, UK) with 10 mm pathlength was used and all measurements were taken under nitrogen flow. Analysis of the spectra was carried out using DichroWeb software, Birkbeck College, University of London, UK [23]. The algorithm which best fit the data was CDSSTR using reference set 4 [24].

### 2.6. Proteolysis of Insulin in dWPI Beads

A SIF solution with 1% (*w/v*) pancreatin (4 × USP) was aliquoted and centrifuged for 30 min at 14,000 rpm at 4 °C. Proteolysis was carried out using a water bath on a hot plate at 37 °C. A beaker containing 25 mL SIF with 3% (*v/v*) of the pancreatin working solution (SIF_+P_) was placed in the bath and stirred at 200 rpm using a paddle stirrer. Once the temperature reached 37 °C, dry insulin-loaded beads were added to the beaker. A sample was immediately withdrawn and replaced with SIF_+P_ to keep enzyme concentrations constant. Samples were withdrawn at 0, 15, 30, 60, 120, 180, and 240 min and added to 150 µL cold HCl (0.1 M) to stop proteolysis. Samples were then filtered using centrifuge filter tubes, stored at −20 °C and analysed by RP-HPLC. The same protocol was carried out with insulin beads in the absence of pancreatin (negative control), while native insulin solution (1 mg/mL) in the presence of SIF_+P_ was used to confirm enzyme activity [25]. A protein assay was carried out on the SIF_+P_ solution to standardise the pancreatin concentration from experiment to experiment. This was performed using a Quant-iT^™^ protein assay kit and according to the manufacturer’s instructions (Thermo Fisher Scientific, Dublin, Ireland). The average pancreatin concentration was 60 ± 6 μg/mL.

### 2.7. Cell-Based Bioactivity Assay for dWPI Bead-Released Insulin

Insulin released from three independent batches of dWPI beads was evaluated for bioactivity in insulin receptor-expressing HepG2 liver cells according to methods described in Santalices et al. [26]. Insulin receptor activation was assessed by transfecting the cells with an insulin-responsive promotor coupled to a luciferase gene. The plasmid was pSynSRE-T-luc, which contained the −324 to −225 bp fragment of the hamster 3-hydroxy-3-methyl-glutaryl-coenzyme A (HMG-CoA) synthase promoter with the Sterol Response Elements (SRE) upstream of the minimal HMG-CoA synthase TATA box (−28 to +39) [27,28]. Insulin regulates HMG-CoA synthase expression through these SRE sites in human cells [29]. A plasmid bearing a four-point mutation in the SRE was transfected in parallel as a negative control (pSynSRE-Mut-T-luc) [29]. A plasmid containing the gene for β-galactosidase (pRVS-βgal) was also transfected alongside the other plasmids to monitor the success of the transfection. The promotor of this gene allows for constitutive expression and should be expressed regardless of the presence of insulin.

HepG2 cells were cultured in Eagle’s Minimum Essential Medium (EMEM) (1 g glucose/L) supplemented with 10% Fetal Bovine Serum (FBS), 1% non-essential amino acids, 2mML-glutamine, 1% penicillin-streptomycin. Cells were passaged once weekly by trypsinization for 5min. MW48 multi-well plates (Costar, New York, NY, USA) were coated with 40 μL/well Type I collagen solution in PBS (100μg/mL) before seeding HepG2 cells at a density of 2.5 × 10^4^ cells/well. Cells were grown in EMEM for 24 h before 25 μL of a transfection mixture was added to each well and incubated for 6 h. The transfection mixture was made up of DNA plasmid (35 ng of the corresponding promoter (pSynSRE-T-luc or pSynSRE-mut-T-luc) + 50ng of pRVS-βgal plasmid per well), Viafect^®^ transfection reagent (1.5 μL/well) and EMEM (23.5 μL/well). After 6h incubation, the wells were washed three times with warmed PBS and 400 μL of culture deprivation media was then added and incubated for 4 h. The deprivation media has most of the same composition as the growth medium, but with 0.5% FBS and supplemented with 2mM of metformin. After 4 h, 100 μL of an insulin sample or a control sample in deprivation media was added to the wells and incubated for a further 20 h. Samples included fresh insulin and insulin released from dWPI beads, as well as a PBS control. After 20 h incubation, wells were washed three times with PBS followed by the addition of 40 μL/well of Passive Lysis Buffer 1× (Promega, Madison, WI, USA) with further incubation for 20 min. Lysates were collected and frozen at −20 °C. For analysis, lysates were split into two and analysed for either luciferase activity or β-galactosidase activity. Luciferase activity is quantified by measuring the luminescence produced by the luciferase catalysed conversion of luciferin to oxyluciferin. 20 μL of lysate was mixed with 40 μL of Luciferase Assay Reagent (Promega, Madison, WI, USA) and analysed following manufacturer’s instructions using the luminometer setting of a Mithras microplate reader (LB940, Berthold, Bad Wildbad, Germany). 20 μL of lysate was then mixed with 180 μL of Z-Buffer (60 mM Na_2_HPO4, 40 mM C, 10 mM KCl, 1 mM Mg_2_SO4, and 50 mM β-mercaptoethanol at pH 7.5) and 40 μL of ONPG (ortho-nitrophenol galactopyranoside) reaction buffer (4 mg/mL *O*-nitrophenyl β-d-galactopyranoside (ONPG) in 100 mM phosphate buffer at pH 7.0) and incubated at 37 °C. The reaction was stopped by adding 75 μL/well of Na_2_CO_3_ and was measured for β-galactosidase activity using at 490 nm using a Mithras microplate reader.

### 2.8. Encapsulator-Generated Prototype Beads

Encapsulators are typically used to produce microparticles from polymer solutions with gelling capacity including alginate, whey, and gelatin [30,31,32]. In the current study, we used a Buchi encapsulator (Flawil, Switzerland) to scale up the process and to create a large uniform batch for the in vivo study. We also wanted to investigate whether the encapsulator could produce smaller beads with a faster release rate that would be suitable for the in vivo study. dWPI was filtered using glass vacuum filtration through 0.22-µm Whatman^®^ filter paper. Insulin (80 mg) was dissolved in HCl (0.1 M) and added dropwise to Trizma^®^ base (0.1 M, pH 9). This solution was combined with dWPI and the process was repeated. The solutions were pooled to create a 40 mL dWPI-insulin mixture containing 4% *w/w* insulin.

An acetate buffer (0.5 M) was used to induce gelation in the encapsulator, along with the addition of 0.4% *w/v* low molecular weight (LMW) chitosan (75–85% deacetylated) to aid the process. The 40 mL insulin-dWPI solution was extruded through a 450 µm vibrating nozzle. The air pressure, frequency of the vibrational unit, and the electrode were adjusted until the extruded solution became a visible chain of droplets. Once this had occurred, beads formed and were collected in the acetate hardening solution over 15 min before being rinsed with water. This was aided by using a nylon filter with 50 µm pores (Biodesign^™^, CellMicroSieves^™^, Fisher Scientific, Waltham, MA, USA). Beads were then placed in glass vials for drying, followed by lyophilisation using an AdVantage Pro^®^ laboratory-scale freeze-dryer (SP Scientific, PA, USA). Glass vials were placed at the centre of the shelf and beads were dried over 3 days according to the parameters in Table 1. Control beads were made in the same manner without insulin. Beads were characterised in terms of size, EE, loading, and release as described above.

### 2.9. In Situ Jejunal Instillations

Adult male Wistar-CRL rats were obtained from the UCD Biomedical Facility, and the Charles River Laboratory, UK, and were housed in a pathogen-free environment with controlled conditions of humidity and temperature under a 12:12 h light/dark cycle with access to laboratory chow and filtered water ad libitum. All procedures were carried out under anaesthesia induced with isoflurane gas (Iso-Vet, 1000 mg/g isoflurane liquid for inhalation (Piramal Critical Care, Middlesex, UK)) at the rate of 5 L/min mixed with 4 L/min O_2_ in an induction box and then maintained at 2–2.5 L/min mixed with 1 L/min via a mask using an anaesthesia vaporising unit (Blease Medical Equipment Ltd., Chesham, UK).

In situ rat jejunal instillations were carried out to assess the lyophilised encapsulator-synthesised insulin-dWPI beads. The method was according to previous descriptions [33,34], but with modifications to accommodate the beads. A midline laparotomy was performed under anaesthesia and the jejunum was identified. A section was tied off at one end using size 4 braided silk sutures and a small incision was made ~6 cm from the sutures. A 20 µL pipette tip was cut and used as a funnel for the beads. The tips were pre-filled with ~9 mg of lyophilised beads, taking care to ensure the beads were not tightly packed inside the tip. The tips were inserted into the jejunum at the incision and beads were manoeuvred through the funnel using the blunt, sealed end of a glass capillary tube. This process was repeated using a second tip to achieve a final instilled insulin dose of 50 IU/kg [35]. The beads were pushed at least ~1 cm from the incision.

Once all the dWPI-insulin beads were inside the loop, the section of jejunum was tied off below the incision to create a closed loop. Then, 200 µL of PBS, or PBS in the presence of the intestinal PE, C_10_ (100 mM), was then injected into the loop using a 30G needle [36]. C_10_ was used here in combination with the beads to aid the passage of released insulin across the gut wall, a strategy that was previously effective for insulin released from another type of nanoparticle prototype construct [34]. The abdominal incision was then closed using skin staples. Glucose readings were taken via the tail vein using a glucometer (Accu-chek Aviva, Roche, UK) every 10 min for 2 h. Retro-orbital blood samples were taken every 20 min for 2 h using a glass capillary tube and collected in a heparinised tube. Samples were stored at 2–8 °C before centrifugation at 6500 g for 5 min. Plasma was collected and stored at −20 °C until analysis by insulin ELISA (Mercodia, Uppsala, Sweden). Animals were euthanised at the end of experiments by intracardiac injection of 0.4 mL pentobarbital (Euthatal^™^, Boehringer-Ingelheim, Pirbright, UK).

Insulin solution, PBS, and insulin ad-mixed with C_10_ were also injected into closed jejunal loops as controls. The latter (insulin with C_10_) data was historical data from our group [37]. This data was obtained with Insuman^®^ (recombinant human insulin, Sanofi, Frankfurt, Germany) instead of Gibco’s recombinant human insulin. The loops were formed as described above for these liquid controls but without the need for a funnel or an incision. To measure the relative pharmacological availability (% PA), and relative bioavailability (% F) one group of animals was dosed with 1 IU/kg insulin by subcutaneous (s.c.) injection. % PA was calculated from the blood glucose levels according to Equation (4) below [37].

(4) $$\%\;\mathrm{PA}=\frac{({\mathrm{AAC}}_{\left(\mathrm{inst}\right)})\left({\mathrm{Dose}}_{\left(s.c.\right)}\right)}{({\mathrm{AAC}}_{\left(s.c.\right)})\left({\mathrm{Dose}}_{\left(\mathrm{inst}.\right)}\right)}\times 100$$

where AAC_(inst.)_ is the area above the glucose curve over the 2 h instillation study, and AAC_(s.c.)_ is the area above the glucose curve after s.c. injection of insulin. Dose_(s.c.)_ is the s.c. dose of insulin, and Dose_(inst)._ is the dose of insulin used in instillations. The PK characteristics were estimated using PKSolver^®^ 2.0 software [38]. Non-compartmental analysis was carried out and the percentage relative oral bioavailability (% F) was calculated as follows (Equation (5)):

(5) $$\%\;F=\frac{({\mathrm{AUC}}_{\left(\mathrm{inst}\right)})\left({\mathrm{Dose}}_{\left(s.c.\right)}\right)}{({\mathrm{AUC}}_{\left(s.c.\right)})\left({\mathrm{Dose}}_{\left(\mathrm{inst}.\right)}\right)}\times 100$$

where the other abbreviations were as for Equation (4) [39].

### 2.10. Histology

Jejunal samples from instillation studies were stored in 10% (*w/v*) formalin for 24 h before being embedded in paraffin wax. Tissue sections (5 μm) were cut on a microtome (Leitz 1512; GMI, Ramsey, MN, USA) and mounted on an adhesive-coated glass slide. Two sections were cut from each sample. Of them, one was stained using haematoxylin and eosin (H&E), while the other was stained with Alcian Blue (AB) and Neutral Red (NR).

### 2.11. Statistical Analysis

EE, loading, and size data were analysed using Student’s unpaired *t*-tests. The bioactivity assay analysis was carried out using one-way ANOVA with Dunnett’s post-test to compare the insulin response to their respective controls (buffer blank) and two-way ANOVA with Bonferroni’s post-test was carried out to compare groups. Results were considered significant if *p* < 0.05 compared to control. Statistical analysis was carried out using GraphPad Prism^®^ Version 5.0 software (San Diego, CA, USA).

## 3. Results

### 3.1. Size, Encapsulation Efficiency, and Loading of Insulin Loaded Whey Beads

**Figure 1:**
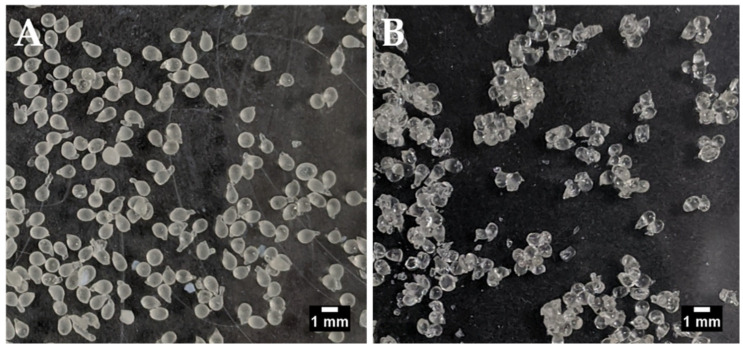
Manually-generated insulin-dWPI beads. (**A**) with Aerosil^®^ drying, (**B**) without Aerosil^®^ drying. Scale bars = 1 mm.

Air-drying led to agglomeration of the beads, but individual beads could still be visualised and were therefore sized manually using ImageJ^®^ software (Figure 1). The Aerosil^®^-dried beads did not agglomerate and were sized using the particle analysis function in ImageJ^®^. The use of Aerosil^®^ to aid the air-drying caused the formation of larger beads (1.49 mm versus 1.04 mm in its absence). This could indicate the presence of Aerosil^®^ on the surface of the beads. The difference in sizing methods may also be responsible to some extent for the size difference between the air-dried and air-dried/Aerosil^®^-treated batches. There were no other significant differences between the batches although, on average, air-drying in the absence of Aerosil^®^ gave a trend for greater insulin loading and EE than in its presence. This is likely because the presence of Aerosil^®^ on the bead surface should increase bead weight while the amount of entrapped insulin should remain the same (Table 2). The EE for insulin in both types of beads was over 60% and the loading values were >2.4%. This is in the context of a theoretical loading of ~4%.

### 3.2. Release of Insulin in Simulated Intestinal Fluids

**Figure 2:**
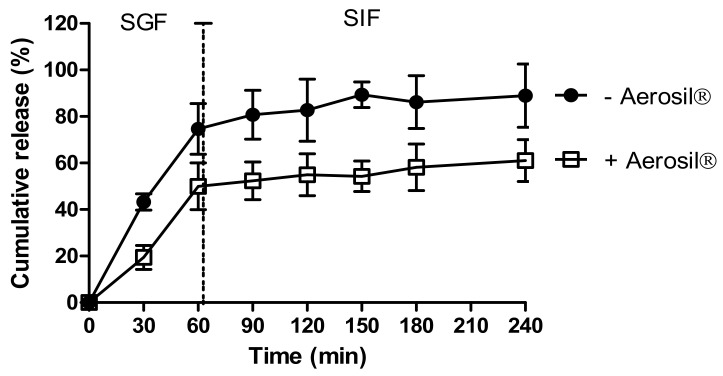
The effect of drying methods on insulin release from dWPI beads. Each point represents mean ± SD (*n* = 3; + Aerosil^®^), (*n* = 2; − Aerosil^®^).

We investigated the effects of Aerosil^®^ on insulin release from dWPI beads. Figure 2 shows the release of insulin from beads air-dried with and without Aerosil^®^ in SGF and SIF. Both bead types released most of the insulin within 60 min in SGF. The absence of Aerosil^®^ allowed ~74% of the insulin to be released in 60 min in SGF, while 50% was released in the presence of Aerosil^®^. This suggests that the Aerosil^®^ may act as a coating, thereby either slowing the release of surface-bound insulin or perhaps by filling the bead pores and reducing insulin release in SGF. Both bead types followed a similar slow pattern of release in SIF, though perhaps for different reasons. 15% additional release was seen in the absence of Aerosil^®^ and 11% release in its presence. The slow-release in SIF in the absence of Aerosil^®^ could be the result of the high percentage of release in SGF, i.e., there was little insulin left to release. In the presence of Aerosil^®^, the slow release in SIF could have been caused by any remaining Aerosil^®^ coating. The Aerosil^®^-coated beads did not fit particularly well with any of the release models (R^2^ adj < 0.9), but the closest fit was the Korsmeyer-Peppas model (Supplementary Table S2). This model was also the best fit for the beads dried without Aerosil^®^. For both types of beads, the *n* value was <0.43, which implies that the release was controlled by diffusion [40]. The swelling study revealed a greater degree of swelling in SGF compared to SIF, suggesting that swelling was a contributing factor to release in SGF (Figure S1).

Air-dried beads exposed to Aerosil^®^ were used for all subsequent characterisation studies as this overall drying method did not cause as much aggregation compared to beads formed in its absence.

### 3.3. Structural Analysis of Released Insulin by CD

**Figure 3:**
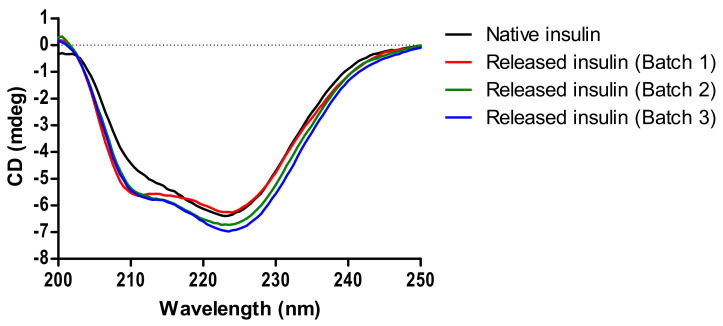
CD spectra of native insulin and bead-released insulin in SIF from three independent batches. Solutions were diluted to 10 µg/mL.

The secondary structure of native insulin in SIF showed characteristic troughs at 211 nm and 222 nm [41]. Bead-released insulin demonstrated a profile similar to native insulin with little differences between three independent batches (Figure 3). Table 3 shows the secondary structure fractions for released insulin and native insulin. This suggests that insulin did not undergo conformational changes during either bead encapsulation or subsequent release.

### 3.4. Degradation of dWPI Bead-Released Insulin by Pancreatin

**Figure 4:**
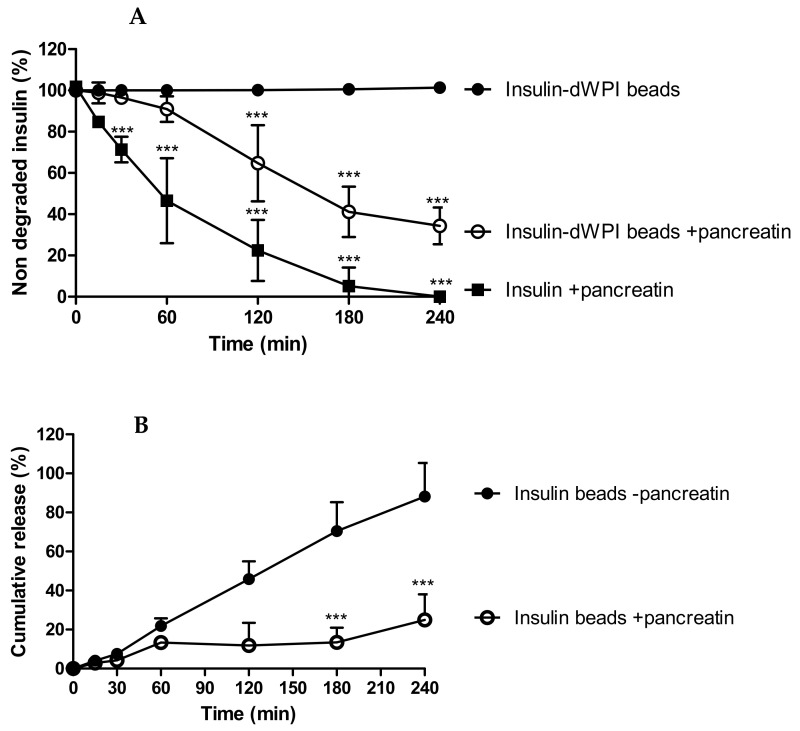
The degradation of insulin released from dWPI beads over 4 h in SIF and SIF supplemented with pancreatin. Data are presented as (**A**) the percentage of insulin remaining following exposure to pancreatin, and (**B**) the effect of pancreatin on the cumulative release of insulin from the dWPI beads. In both cases, insulin released in the absence of pancreatin was used as a control. Native insulin (1 mg/mL) was used to confirm enzyme activity. (Mean ± SD, *** *p* < 0.001 compared to insulin beads in the absence of pancreatin, (*n* = 3).

We determined whether dWPI beads provided protection for entrapped insulin against serine proteases. Native insulin (1 mg/mL) degraded by 50% in 60 min in the presence of pancreatin in SIF, which confirmed the activity of the pancreatic enzymes (Figure 4A). Native insulin was used at a higher concentration (1 mg/mL) than insulin loaded in beads (100–150 µg/mL) because 1 mg/mL was an appropriate concentration to see degradation by pancreatin over 4 h. This is because it occurs within minutes at lower concentrations of the enzyme mixture. The bead concentration was the same concentration used for the release study, so we calculated that it should be detectable at each timepoint. Insulin released from beads in the absence of pancreatin was used as a negative control and from this, we determined the net degradation of insulin from dWPI beads at each time point.

The proteolysis data suggests that the beads provided partial protection against degradation for at least 60 min (i.e., 65% of the released insulin remained (Figure 4B)), after which degradation was apparent because there was significantly less insulin present from 120–240 min compared to the negative control (Figure 4A). In Figure 4A, between 180 and 240 min, degradation of insulin from beads showed signs of reaching a plateau and then insulin release was further increased (Figure 4B). Since dWPI has been reported to be a peptidase inhibitor [14], and this likely occurs through competitive inhibition, perhaps the dWPI from the beads interacted with pancreatin to reduce its capacity for insulin binding at 180 min. The degradation of dWPI by pancreatin could then have reduced the integrity of the bead and allowed more insulin to be released, which was left intact because the enzymes had already reached their binding capacity.

### 3.5. Bioactivity of dWPI-Released Insulin in Transfected HepG2 Cells Expressing Human Endogenous Insulin Receptors

The bioactivity of insulin following encapsulation was examined in insulin receptor-expressing HepG2 cells by measuring the induction of an insulin target gene promoter, HMGCoA synthase. The SRE response element within the promoter drove the expression of luciferase (WT) in our transient transfection assay. As a negative control, a similar plasmid bearing four point mutations in the promoter (MUT) was used. To normalize each well we co-transfected cells with a plasmid bearing a strong promoter (CMV) driving β-galactosidase expression. Results show the intensity ratio between luciferase and β-galactosidase expression (Luc/β-gal) in response to dWPI-bead-released insulin compared to native insulin solution. The results were normalised against the negative control (buffer blank). Three independent batches of insulin-loaded beads were made, and the insulin was released in PBS. The bioactivity data for each batch were pooled, as there was little variation in Luc/β-gal expression between batches.

**Figure 5:**
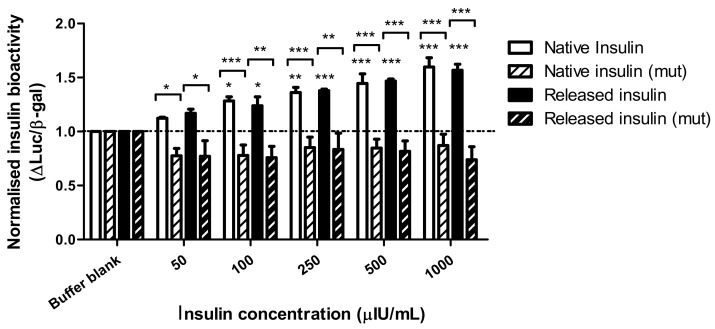
The ratio of luciferase and β-galactosidase expression in endogenous insulin receptor-expressing HepG2 cells transfected with wt (SynSRE-T-Luc) or mutant (mut, SynSRE-T-mut-Luc), following exposure to native and bead-released insulin. Data are presented as mean ± SEM, *n* = 3 individual bead batches. * *p* < 0.05, ** *p* < 0.01, *** *p* < 0.001 compared to buffer blank. Bracketed values compared responses from enriched and mutant cells.

Figure 5 shows that Luc/β-gal expression was induced by both native and bead-released insulin at 50 µIU/mL and above. There was also no statistical difference between the Luc/β-gal response induced by native insulin compared to insulin released from the dWPI beads. There was no induction of the mutant promotor at the insulin concentrations tested, thereby demonstrating a specific action of insulin on the SRE for luciferase expression. Since the bead-released insulin could induce a luciferase response similar to the native insulin by activating endogenous insulin receptors on HepG2 cell plasma membranes, this confirms the CD data and allows a conclusion that no structural changes occurred during the bead production process that might prevent insulin from binding to the promotor to cause a functional response. The bead synthesis and drying processes are therefore gentle enough to allow insulin bioactivity to be retained in vitro.

### 3.6. Characterisation of Prototype Insulin-dWPI Beads Made by the Buchi Encapsulator

**Figure 6:**
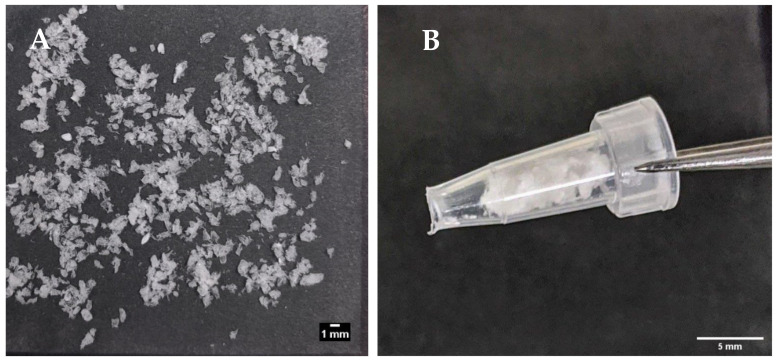
(**A**) Encapsulator-generated, lyophilised insulin-dWPI beads; (**B**) the same beads packed inside a funnel for the in vivo rat jejunal instillation study.

**Figure 7:**
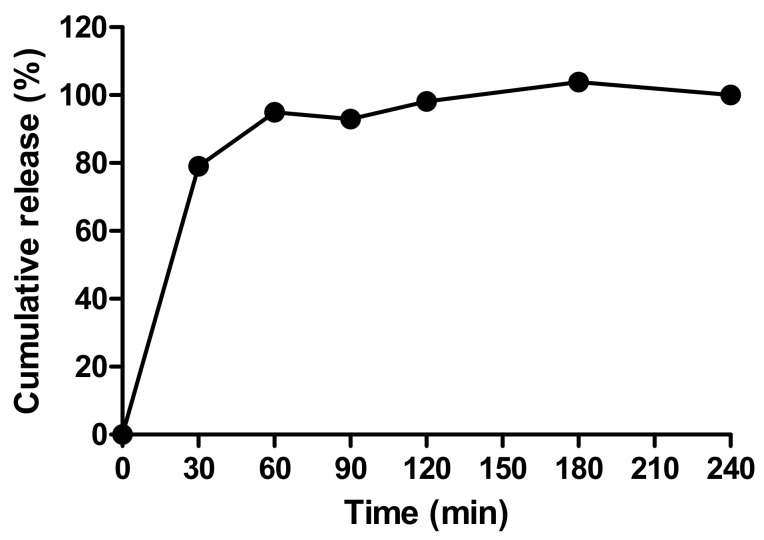
The release profile of insulin released from prototype beads in PBS.

Figure 6A shows the prototype beads made by the encapsulator. These were slightly larger than the manually-generated beads (1.66 vs. 1.49 mm), though any aggregates produced by the encapsulator process were easily dispersed and the beads fitted inside the delivery funnel for i.j. administration (Figure 6B). The EE for the prototype was 42% possibly due to insulin adsorption to tubing connect to the encapsulator nozzle. This creates a larger surface area for adsorption compared to the manual bead production method. Nonetheless, the insulin loading of 2.77% was similar to that of the manually-produced beads, and from this, it was calculated that ~17.5 mg of the prototype would be needed for i.j. administration to a 280 g rat to achieve an insulin dose of 50 IU/kg (Table 4). Insulin released quickly from the lyophilised prototype beads in PBS, with ~95% released in 60 min, and 100% released between 120–180 min (Figure 7). The release model with the best fit was the first order kinetics model, though the Korsmeyer-Peppas model was also a good fit (Supplementary Table S2). The first-order model describes drug release where the release is proportional to the drug remaining in the matrix and is concentration-dependent [42]. For the Korsmeyer-Peppas model, *n* < 0.43, which would suggest diffusion as a release mechanism in spherical particles. The release pattern of the prototype likely models the behaviour that would be exhibited in this in vivo model, as PBS is injected into the jejunal loops after the beads.

### 3.7. In Vivo Performance of Insulin-Loaded Prototype dWPI Beads: Rat Jejunal Instillations

**Figure 8:**
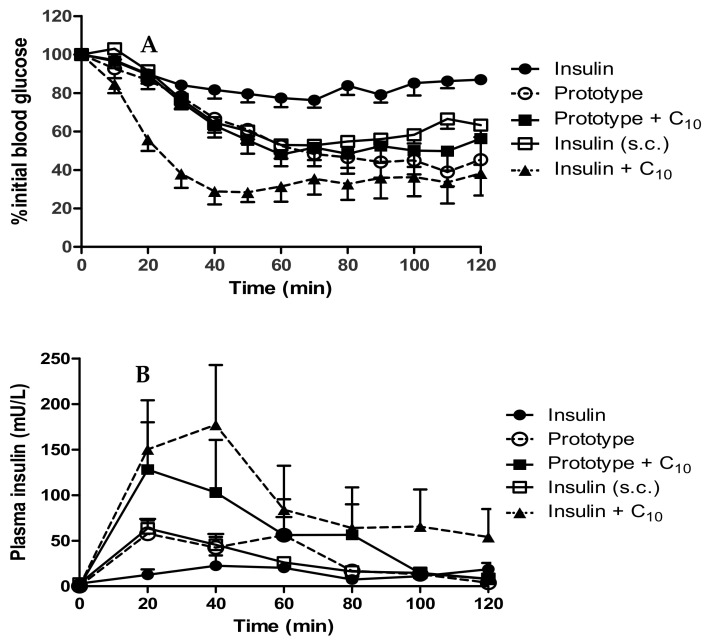
(**A**) Reduction in blood glucose; (**B**) Plasma insulin levels in rats following i.j. instillation of prototype dWPI-insulin beads with and without C_10_. Doses: 50 IU/kg insulin, C_10_ (100 mM) (both i.j.) and s.c.-administered insulin (1 IU/kg). Data presented as mean ± SEM, *n* = 4–6.

Beads administered by the i.j. route to rats led to a decrease in plasma glucose levels to 45.3 ± 6.7% of the initial blood glucose (T0) in 60 min at a dose of 50 IU/kg (Figure 8A). This was similar to the response produced by s.c.-administered native insulin (54.3 ± 5.2% of T0) at a dose of 1 IU/kg (albeit at a 50-fold higher dose), but a far greater reduction than that induced by native insulin administered by the i.j. route. The inclusion of C_10_ (100 mM), with dWPI-insulin beads, did not induce any further reduction in blood glucose levels (49.1 ± 6.2% of T0). Inclusion of historical data in which C_10_ was ad-mixed with native insulin and administered by the i.j. route shows the drop in blood glucose when the two were combined in this rat model [37]. The % PA calculated for the insulin-dWPI bead prototype, and the prototype + C_10_ was 2.4% and 2.3% respectively (Table 5). The % PA for native insulin admixed with C_10_ from our historical data was 2.5% [32].

The prototype increased plasma insulin slightly more by the i.j. route than the s.c.-administered insulin (C_max_ of 87.4 mU/L compared to 64.8 mU/L). Instead of decreasing over time in the pattern of the s.c.-administered insulin, the insulin plasma level from the prototype increased again at 60 min (Figure 8B). This could be the result of the continuous insulin release from the beads which reached ~100% by 60 min (Figure 7). The prototype reached T_max_ later than the s.c.-administered insulin (36.7 min compared to 23.3 min), likely because the insulin had to be released from beads before it was available for absorption. The addition of C_10_ to the beads produced variable results following i.j. instillation. The C_max_ (128.9 mU/L) was higher on average than for the prototype, and the T_max_ was earlier (25 min), which suggested that C_10_ increased the intestinal absorption of insulin. The insulin + C_10_ ad-mixture data was also variable but on average had a later T_max_ (43.3 min) and a higher C_max_ (215 mU/L) than the prototype + C_10_. The data were statistically different from i.j.-instilled insulin at the 20- and 40-min time points. The % relative F calculated for the prototype was 2.2%, and the presence of C_10_ increased this to 4.3% (Table 5). The data for insulin + C_10_ reported a% F for insulin of 3.3% [37].

**Figure 9:**
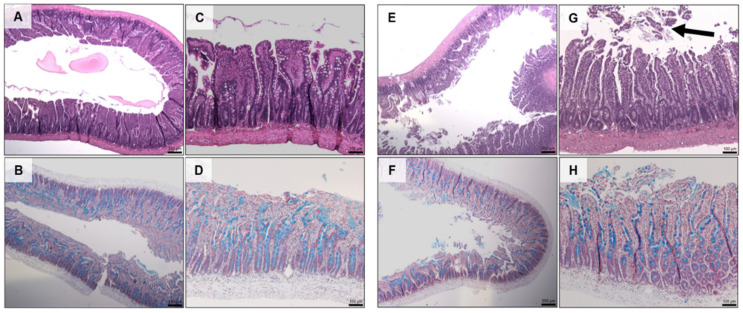
H & E (top) and AB & NR (bottom) staining of jejunal tissue following 2 h in vivo exposure to dWPI-insulin beads with and without C_10_. (**A**–**D**) prototype, (**E**–**H**) prototype + C_10_. Scale bars = 250 µm (**A**,**B**,**E**,**F**), and 100 µm (**C**,**D**,**G**,**H**). Arrow in **G** shows cell sloughing.

Histology showed that there was no damage to jejunal tissue when exposed to the prototype for 120 min compared to untreated controls (Figure 9C). The addition of C_10_ caused mild erosion of the epithelium and cell sloughing (Figure 9G), but there was no change in mucus production (Figure 9D vs. Figure 9H).

## 4. Discussion

The proportion of diabetics worldwide is expected to increase by 51% by 2045 [43], of which the majority are Type II. New treatment options are needed to better manage blood glucose levels which will, in turn, reduce diabetic complications and improve prognoses. Oral insulin would be a more convenient option for patients who are non-compliant with current s.c. administrations, while also reducing the unwanted side effects of s.c.-administered insulin, and encouraging them psychologically to be able to opt for it earlier in the disease [44]. Overall, this could improve the quality of life for many patients.

This study sought to incorporate insulin in dWPI beads to determine the initial suitability of the system for both insulin and other peptides that might also be considered for oral administration. It then expanded on this by scaling up the process and investigating in vivo efficacy in a rat intestinal instillation in vivo model. We showed that dWPI beads can entrap insulin and partially protect it from enzymatic degradation. The beads had reasonable insulin loading (~25 µg/mg/2.5%) considering that the theoretical loading was 4%. The values were, however, lower than those reported for some other insulin delivery systems using other food-sourced natural polymers, including alginate, chitosan, and carrageenan [45,46]. Substantial insulin release from dWPI beads in SGF was observed, but this is not considered an unsurmountable issue as the formulation would likely be administered in an enteric-coated capsule where it can release in the jejunum [47]. We found that the use of Aerosil^®^ to assist in air-drying the beads also had a surprising benefit for desired insulin release characteristics. A comparison between insulin release with and without Aerosil^®^ as the drying agent revealed that Aerosil^®^ reduced the overall release rate in SGF. Trace amounts of Aerosil^®^ were expected to wash off the beads as soon as the beads were immersed in the solution, but instead, the fumed silica seems to have created a barrier on the bead surface.

We also demonstrated that the formulation process was suitably mild for use with a peptide and did not alter the secondary structure or function of insulin following receptor binding. This was shown by the bioactivity assay in HepG2 cells expressing endogenous human insulin receptor levels where the ratio of Luc/β-gal increased with increasing concentrations of insulin, indicating the preservation of insulin bioactivity following release from the beads. Our encapsulated dWPI insulin was also active at physiological ranges found in human serum [48]. This assay is a good alternative to in vivo bioactivity studies and provides important information that would prevent inactivated insulin formulations from being prematurely transferred for in vivo studies. The assay was used in the same way recently as a screening tool to enable subsequent rat intestinal instillation studies of two other formulations of insulin under consideration for oral delivery [25,26].

While the manually-generated beads achieved a reasonable insulin loading for the formulation, the level obtained would have equated to 21 mg of material (i.e., ~54 beads) to reach the required insulin dose of 50 IU/kg for a 300 g rat. It would not have been practical to administer this number of beads in an in vivo setting, which is what prompted the use of an encapsulator. The encapsulator-generated beads were of a similar size to the manually generated beads, but due to lyophilisation, they were easier to manipulate into the delivery funnel to instil into jejunal loops. Higher loading of insulin (~28 µg/mg) in the encapsulator-generated beads reduced the amount of material required for the in vivo study from 21 mg to ~17 mg per rat. Lyophilisation also hastened the release of insulin from the encapsulator beads in PBS. This confirmed our observation that the drying method contributes to bead characteristics. Freeze-drying likely increased the porosity of the beads thereby facilitating release. This has been observed before by Fonte et al. who studied the effect of cryoprotectants on the porosity of lyophilised poly(lactic-co-glycolic acid) (PLGA) nanoparticles [49]. Further optimisation of the encapsulation process and the formulation could improve EE and loading of insulin. It may potentially also reduce the bead size, as the encapsulator can create smaller particles by using nozzles of a smaller diameter. A 150 µm nozzle was used by Doherty et al. for example, to create probiotic-loaded microbeads from dWPI using a similar encapsulator (Inotech^®^, Greifensee, Switzerland) [50].

The target attributes for the prototype insulin-dWPI formulation were to improve upon the insulin loading >2.5% seen with the manually-produced beads, quick release in PBS (~100% within 60 min), and a size that would facilitate i.j. administration via a delivery funnel. It has been observed in previous studies that oral insulin particulate formulations often need additional help from PEs to enable released peptide to cross the epithelium [34,51]. It was for this reason that an established PE, C_10_, was tested in combination with the insulin-loaded beads. This is also the reason why a quick release of insulin was desirable in SIF. Insulin needs to be present at the small intestinal epithelium at the same time that the PE interacts with the epithelium because the permeation-enhancement effect of agents such as C_10_ is local and transient [52,53].

Nonetheless, the dWPI-insulin beads alone were effective at reducing blood glucose and further addition of C_10_ did not improve the PD effect any further. The Cmax and % F were higher when C_10_ was administered with the insulin-dWPI beads compared to the beads without C_10_, but values were significantly different at 20 min. The 100 mM concentration of C_10_ that was present in the jejunal loops was also enough to independently reduce plasma glucose, as was also documented in ad-mixtures with insulin in rat intestinal instillations in other studies [37,51]. This response was rapid and occurred within 10–20 min [51]. This would suggest that the bead-released insulin was not able to take full advantage of the PE action of C_10_ using the current study design. The reduction in blood glucose produced by the dWPI-insulin beads may have been facilitated by a potential PE action of dWPI itself [18]. Finally, it cannot be ruled out that CS may also have been present on the beads as it was used to aid gelation. CS could have aided insulin absorption as it is known to possess PE and mucoadhesive properties [54]. Further studies are needed to clarify whether insulin permeability across the rat intestine was aided by bead constituents, CS or dWPI, or both.

## 5. Conclusions

As an oral peptide delivery system, dWPI beads have promising characteristics and were well tolerated in rat intestinal instillations in vivo. With further formulation adjustments to maximise peptide loading and reduce bead size, along with enteric coatings, dWPI beads could be a viable option for oral peptide and macromolecule delivery.

## Figures and Tables

**Table 1:** Lyophilisation process parameters used to prepare prototype beads.

	Shelf Temp (°C)	Ramp Rate (°C)	Hold Time (min)	Vacuum (mbar)
Loading	5	0.5	30	~700
Freezing	−40	0.5	300	~700
Primary drying	−30	0.3	2800	0.13
Secondary drying	25	0.3	240	0.13
Storage	5	NA	NA	800

**Table 2:** Size, EE, and loading of air-dried insulin loaded-beads, with or without Aerosil^®^. Data presented as mean ± SD, *n* = 2–3.

Air-Dried Beads	Diameter (mm)	EE (%)	Loading (%)	Loading (µg/mg Beads)
+ Aerosil^®^	1.49 ± 0.27 ***	61.42 ± 11.01	2.46 ± 0.34	24.65 ± 3.38
− Aerosil^®^	1.09 ± 0.13	72.59 ± 21.26	3.22 ± 0.40	32.78 ± 4.84

*** *p* < 0.001.

**Table 3:** The secondary structure fractions as calculated by CDSSTR^®^ software.

	Helix 1	Helix 2	Strand 1	Strand 2	Turns	Unordered
Native insulin	0.01	0.05	0.24	0.12	0.23	0.33
Released insulin (Batch 1)	0.03	0.09	0.17	0.11	0.26	0.33
Released insulin (Batch 2)	0.03	0.09	0.17	0.11	0.26	0.34
Released insulin (Batch 3)	0.03	0.09	0.16	0.11	0.27	0.34

**Table 4:** Characterisation of insulin-dWPI beads synthesised by the Buchi encapsulator. For diameter, the data are presented as mean ± SD across 73 individual bead measurements.

	Diameter (mm)	EE (%)	Loading (%)	Final Loading (µg/mg)	Beads (mg) per Rat
Prototype beads	1.66 ± 0.89	42.02	2.77	27.71	17.53

**Table 5:** PK-PD parameters from i.j. instillations with prototype insulin-dWPI beads (mean ± SD, *n* = 4−6). Insulin + C_10_ (100 mM) is historical data and, as such, the % F and % PA were calculated using a different s.c. dataset ([39]).

	T_max_ (min)	C_max_ (mU/L)	AUC (mU/L.min)	% PA	% F
Insulin (s.c.)	23.3 ± 8.2	64.9 ± 22.7	3356 ± 995	-	-
Beads	36.7 ± 19.7	87.4 ± 38.8	3761 ± 1429	2.4	2.2
Beads + C_10_	25.0 ± 10.0	128.9 ± 105.4	7239 ± 6675	2.3	4.3

## Data Availability

The data presented in this study are available on reasonable request from the corresponding author.

## Supplementary Materials

The following are available online at https://www.mdpi.com/article/10.3390/pharmaceutics13050656/s1, Table S1: The formulas used for each mathematical model of drug release. Table S2: The R2 adj and other parameters calculated for each type of release model. Figure S1: Swelling behaviour of insulin loaded beads.
