# Supplementary material for: Synthesis and In Vivo Evaluation of Insulin-Loaded Whey Beads as an Oral Peptide Delivery System
Source: Pharmaceutics. 2021 May 4;13(5):656. doi: 10.3390/pharmaceutics13050656 (PMC8147814; doi:10.3390/pharmaceutics13050656)
Supplement: Supplementary file 1 [file pharmaceutics-13-00656-s001.zip › pharmaceutics-1209153-supplementary.pdf]

pharmaceutics

MDPI

Supplementary Materials: Synthesis and In Vivo Evaluation of Insulin-Loaded Whey Beads as an Oral Peptide Delivery System

Joanne Heade, Fiona McCartney, Miguel Chenlo, Olga Moreno Marro, Maja Severic, Robert Kent, Sinead B. Bleiel, Clara V. Alvarez, Brendan T. Griffin and David J. Brayden

S1. Release modelling

Table S1. The formulas used for each mathematical model of drug release. F = fraction (%) of drug released over time (t). Adapted from Zhang *et al.* [22].

Model	Formula	Parameters
**Zero Order**	$F = k_0(t)$	k_0 = zero order release constant
**First order**	$F=100(1-e^{-k1(t)})$	k_1= first order release constant
**Higuchi**	$F = k_H(t^{0.5})$	k_H = Higushi release constant
**Korsmeyer-Peppas**	$F=k_{KP}(t^n)$	k_{KP} = release constant incorporating structural and geometric characteristics of the drug-dosage form<br>n = diffusional exponent

Table S2. The R^2adj and other parameters calculated for each type of release model applied to the SGF/SIF release data. Underlined and shaded values indicate the best fit.

	Parameters	+ Aerosil®	- Aerosil®	Prototype
**Zero order**	k_0	0.345	0.525	0.606
	R^2adj	0.3885	0.1713	−0.4055
	AIC	62.9117	71.1550	67.3616
**First order**	k_1 (min^{-1})	0.006	0.018	0.050
	R^2adj	0.755	0.932	<u>0.993</u>
	AIC	55.591	46.851	29.903
**Higuchi**	k_H	4.578	7.031	8.427
	R^2adj	0.8630	0.8309	0.6057
	AIC	50.8879	58.4333	58.4639
**Korsmeyer-Peppas**	*kKP*	10.166	23.080	57.735
	n	0.344	0.259	0.108
	R^2adj	<u>0.889</u>	<u>0.9375</u>	0.989
	AIC	49.929	51.251	34.119

S2. Swelling behaviour

Insulin beads were weighed and placed into a series of Eppendorf tubes (~10 mg/tube). The tubes were labelled T0, T15, T30, T60, T90, and T120. 1 mL SGF was added to one set of tubes and 1 mL SIF was added to a second set. Both sets of Eppendorf tubes were incubated on a shaker at 600 rpm at 37 °C for 2 h (Titramax 1000, Heidolph, Germany). T0 samples were immediately removed, the SGF/SIF was discarded, and the beads were placed on a paper towel before being weighed. This was repeated for all time points. The degree of swelling was calculated according to the equation below.

$$Degree\ of\ Swelling\ (\%) = \frac{Weight\ at\ Tx - Initial\ weight\ (mg)}{Initial\ weight\ (mg)}$$

Where Tx is the various time points, and the initial weight is the known weight of beads added to that particular tube at the start of the study. The swelling calculation for T0 samples was subtracted from the other time points to account for any surface water that was not removed by the paper towel.

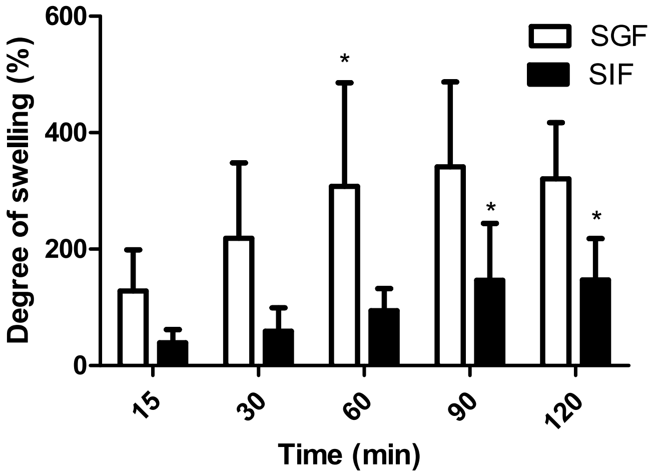

Figure S1. Swelling behaviour of insulin loaded beads after drying with Aerosil® followed by incubation in SGF (pH 1.2) and SIF (pH 6.8). Data are presented as mean ± SD, * $p < 0.05$ compared to T0, with $n = 3$. The swelling was particularly evident in SGF.
